# Whole-Genome Gene Expression Profiling of Formalin-Fixed, Paraffin-Embedded Tissue Samples

**DOI:** 10.1371/journal.pone.0008162

**Published:** 2009-12-03

**Authors:** Craig April, Brandy Klotzle, Thomas Royce, Eliza Wickham-Garcia, Tanya Boyaniwsky, John Izzo, Donald Cox, Wendell Jones, Renee Rubio, Kristina Holton, Ursula Matulonis, John Quackenbush, Jian-Bing Fan

**Affiliations:** 1 Illumina, Inc., San Diego, California, United States of America; 2 Expression Analysis, Inc., Durham, North Carolina, United States of America; 3 Department of Biostatistics, Dana-Farber Cancer Institute, Boston, Massachusetts, United States of America; 4 Department of Medical Oncology, Dana-Farber Cancer Institute, Boston, Massachusetts, United States of America; Duke-NUS Graduate Medical School, Singapore

## Abstract

**Background:**

We have developed a gene expression assay (Whole-Genome DASL®), capable of generating whole-genome gene expression profiles from degraded samples such as formalin-fixed, paraffin-embedded (FFPE) specimens.

**Methodology/Principal Findings:**

We demonstrated a similar level of sensitivity in gene detection between matched fresh-frozen (FF) and FFPE samples, with the number and overlap of probes detected in the FFPE samples being approximately 88% and 95% of that in the corresponding FF samples, respectively; 74% of the differentially expressed probes overlapped between the FF and FFPE pairs. The WG-DASL assay is also able to detect 1.3–1.5 and 1.5–2 -fold changes in intact and FFPE samples, respectively. The dynamic range for the assay is ∼3 logs. Comparing the WG-DASL assay with an *in vitro* transcription-based labeling method yielded fold-change correlations of R^2^ ∼0.83, while fold-change comparisons with quantitative RT-PCR assays yielded R^2^∼0.86 and R^2^∼0.55 for intact and FFPE samples, respectively. Additionally, the WG-DASL assay yielded high self-correlations (R^2^>0.98) with low intact RNA inputs ranging from 1 ng to 100 ng; reproducible expression profiles were also obtained with 250 pg total RNA (R^2^∼0.92), with ∼71% of the probes detected in 100 ng total RNA also detected at the 250 pg level. When FFPE samples were assayed, 1 ng total RNA yielded self-correlations of R^2^∼0.80, while still maintaining a correlation of R^2^∼0.75 with standard FFPE inputs (200 ng).

**Conclusions/Significance:**

Taken together, these results show that WG-DASL assay provides a reliable platform for genome-wide expression profiling in archived materials. It also possesses utility within clinical settings where only limited quantities of samples may be available (e.g. microdissected material) or when minimally invasive procedures are performed (e.g. biopsied specimens).

## Introduction

Formalin-fixed, paraffin-embedded (FFPE) tissues represent an invaluable resource for cancer research, as they are the most widely available material for which patient outcomes are known. There were over 300 million archived cancer tissue samples in the United States in 1999, with more samples accumulating at a rate of over 20 million per year [1]. The ability to perform gene expression profiling in these samples will enable both prospective and retrospective studies, and should greatly facilitate research in correlating expression profiles with clinical outcomes [2]. However, formalin fixation is known to render adenosine residues particularly prone to chemical modifications such as methylene dimerization and mono-methylolation [3] and generate degraded RNA fragments (up to 50% of which may not contain a poly-A tract) [4]. The degradation and chemical modification of RNA during tissue fixation and storage present challenges when applying conventional microarray technologies [3], [5]–[8].

To overcome the technical limitations to microarray-based analyses of FFPE samples, we previously developed a sensitive and reproducible gene expression profiling assay, DASL (cDNA-mediated annealing, selection, extension and ligation), for parallel analysis of hundreds of genes with highly degraded RNA samples [9]–[11]. The DASL assay incorporates random priming during cDNA synthesis, and therefore does not depend solely on poly-A/oligo-dT based priming. In addition, the assay requires a relatively short target sequence of about 50 nucleotides for query oligonucleotide annealing; thus, it can effectively quantify degraded RNA samples. While most other technologies rely on multiple rounds of random priming for sample amplification and labeling [3], DASL only generates first strand cDNA that minimizes variation that arises during the random priming. This technology has been successfully used to profile a variety of archived FFPE tumor samples, some of which have been in storage for as long as 24 years [12] and for which, in many cases, little or no tissue handling and fixation details are known, including colon [10], [11], breast [10], [11], [13]–[16], lung [11], [14], prostate [11], [12], [17]–[19], bladder [16], and liver [20] cancer. While all of these applications clearly demonstrated the utility of the technology for identification and validation of tissue and cancer-specific markers, the DASL assay was limited by the number of genes that could be profiled simultaneously (up to 1536 targets).

In this study, we extended the assay to include all 24K well-annotated RefSeq genes. We provide here a comprehensive characterization of the assay sensitivity and quantitative performance on FFPE tissues. This opens up new avenues for large-scale discovery, validation, and clinical applications of mRNA biomarkers in human diseases.

## Results

### Assay Reproducibility

**Figure 1:**
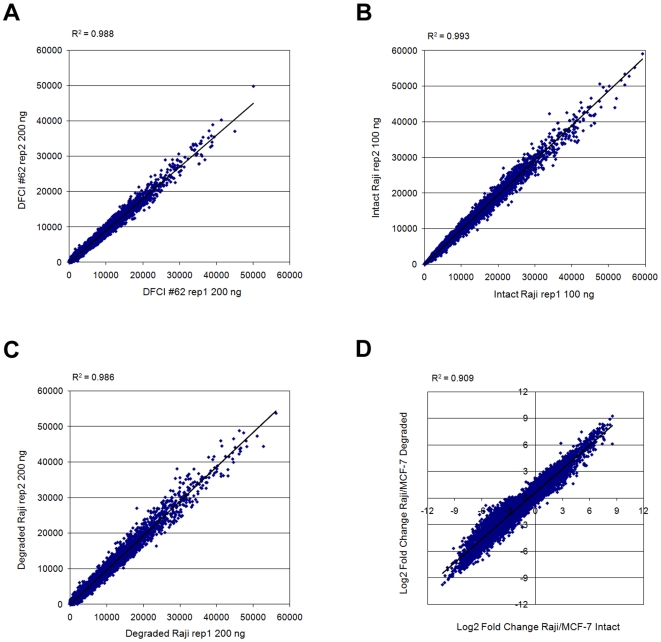
WG-DASL assay reproducibility with variable RNA inputs. Raw intensity scatterplots (all 24526 probes) are shown for WG-DASL assay replicates (replicate 1, x-axis; replicate 2, y-axis) for (A) 200 ng total RNA derived from a formalin-fixed, paraffin-embedded (FFPE) ovarian tumor; (B) 100 ng intact Raji RNA; and (C) 200 ng artificially degraded Raji RNA; (D) The fold-change correlation between intact (x-axis) and artificially degraded (y-axis) cell line RNAs was calculated as the Log_2_ of the fold-change between the Raji and MCF-7 sample intensities. All detected probes (Detection p-value <0.01) are plotted.

To assess the reproducibility of the WG-DASL assay we initially used inputs of 200 ng total RNA extracted from six different ovarian tumor FFPE tissues. The samples were labeled and hybridized as described in the Materials and Methods section. Each sample was assayed twice and the correlation was assessed for each FFPE tissue. Raw intensity scatterplots of a representative example, yielded a correlation coefficient R^2^ = 0.988 and a slope of approximately 45 degrees (Fig. 1A). When probe detection concordance rates (Detection p-value <0.01) were calculated between replicates, a high degree of overlap was obtained, with an average probe concordance rate of 94.6%. Detailed data for two representative FFPE samples are presented in Table 1.

**Table 1:** WG-DASL Assay Performance as a Function of FFPE RNA Input.

Metric	200 ng	50 ng	10 ng	5 ng	1 ng	250 pg
Average number of detected probes (DFCI #120)	19066	18393	18152	17627	12742	6079
Average number of detected probes (DFCI #129)	18031	17677	17113	16404	10998	5585
Average probe overlap with 200 ng (DFCI #120; %)	100.0	93.6	91.0	88.7	63.7	30.8
Average probe overlap with 200 ng (DFCI #129; %)	100.0	95.7	90.8	87.3	60.5	30.8
Average correlation with 200 ng (DFCI #120; R^2^)	100.0	0.951	0.920	0.895	0.794	0.636
Average correlation with 200 ng (DFCI #129; R^2^)	100.0	0.957	0.903	0.873	0.748	0.398
Self-Reproducibility (DFCI #120; R^2^)	0.987	0.965	0.959	0.935	0.800	0.724
Self-Reproducibility (DFCI #129; R^2^)	0.986	0.979	0.958	0.940	0.803	0.680

Values for the number of detected probes, probe overlap and correlation are derived from the average of two technical replicates. Probe overlap is calculated as a percentage of the number of probes with matching detected calls at p-value <0.01 between the low and standard inputs divided by the total number of probes detected in the standard input (200 ng). Values shown for the correlation and self-reproducibility are calculated for probes detected (p <0.01) in both inputs and replicates, respectively.

Profiling of two intact (100 ng) and artificially degraded (200 ng; 95°C for 30 min) cancer cell line RNAs (Raji and MCF-7) also yielded highly reproducible expression profiles, with an average correlation of R^2^∼0.989 (Fig. 1B, Table 2) and 0.985 (Fig. 1C, Table 2) for the intact and artificially degraded samples, respectively. Average probe concordance rates between replicates were 97.9% and 95.9% for the intact and artificially degraded samples, respectively (Table 2). Despite the relatively high degree of overlap of detected probes between the intact and the corresponding artificially degraded samples (∼93.9%), direct comparisons of the raw intensities between intact and artificially degraded samples yielded correlations of R^2^∼0.71. This is thought to be due to different degradation rates and/or secondary structures of mRNAs between intact and degraded samples [4], [21]. Therefore we performed fold-change comparisons in which intact sample pairs were compared to their corresponding degraded samples pairs. Using this approach highly correlated data were obtained, with an average R^2^ = 0.91 (Fig. 1D). This result suggests that our method can faithfully detect differentially expressed genes in intact as well as degraded samples.

**Table 2:** WG-DASL Assay Performance with Intact and Artificially Degraded RNA Inputs.

Metric	Intact MCF-7	Intact Raji	Degraded MCF-7	Degraded Raji
Average number of detected probes	15247	13291	14535	12675
Average probe overlap with intact RNA (%)	100.0	100.0	93.5	94.2
Probe concordance (%)	97.6	98.2	94.8	97.1
Self-Reproducibility (R^2^)	0.988	0.990	0.982	0.988

Values for the number of detected probes and probe overlap are derived from the average of two technical replicates. Probe overlap is calculated as a percentage of the number of probes with matching detected calls at p-value <0.01 between the artificially degraded and intact inputs divided by the total number of probes detected in the intact samples. Values shown for self-reproducibility are calculated for probes detected (p <0.01) in both replicates. Artificially degraded RNA was generated by heating intact RNA at 95°C for 30 min.

### Comparison between Fresh-Frozen and FFPE Samples

**Figure 2:**
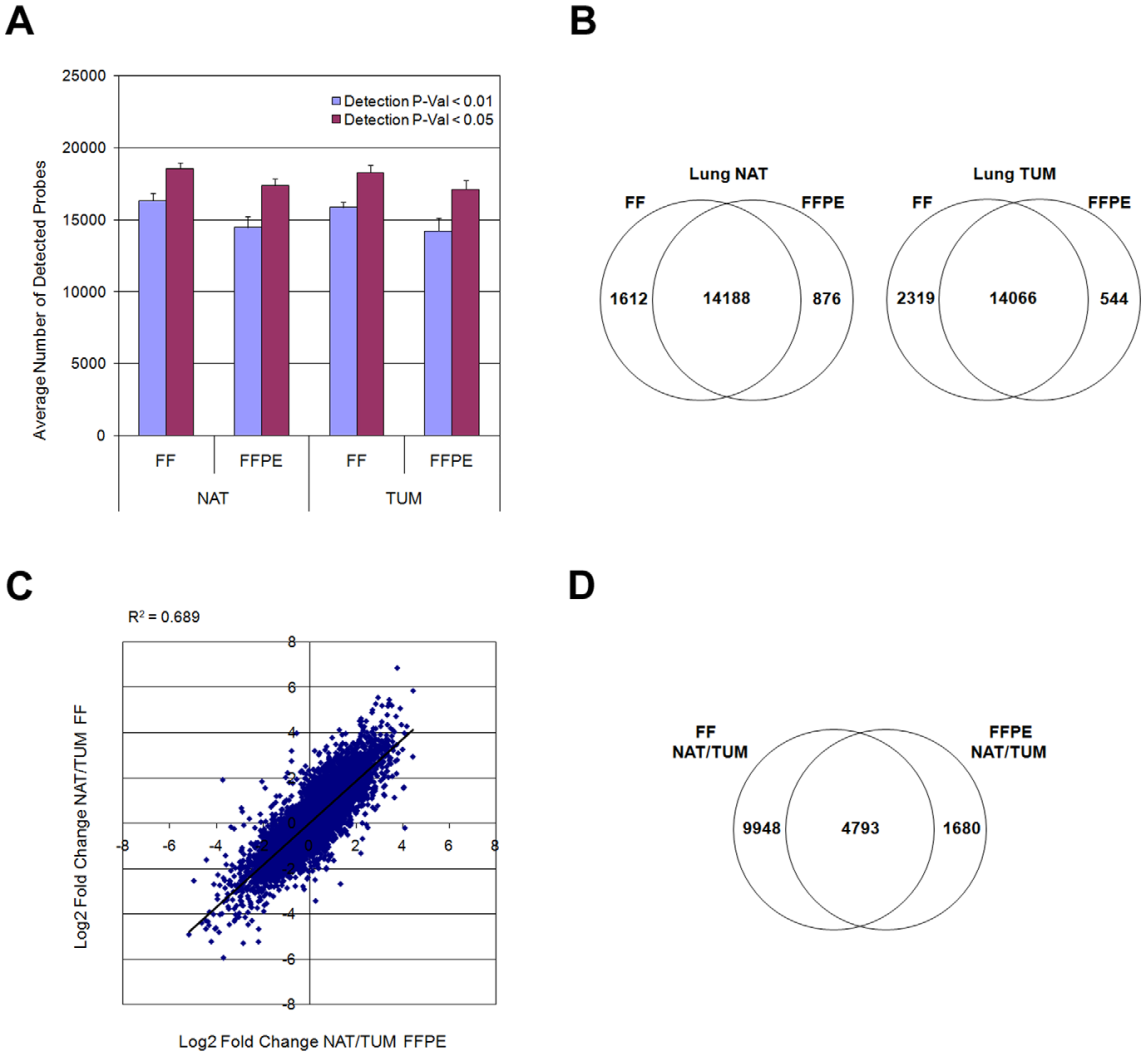
Comparison between fresh-frozen (FF) and FFPE samples. Matched FF and FFPE samples for lung tumor (TUM) and normal adjacent tissue (NAT) were labeled and hybridized using the WG-DASL assay. (A) Assays were performed in quadruplicate and the average number of detected probes plotted for each sample (error bars ± SEM). (B) The overlap of detected probes between the matched FF and FFPE samples for both the NAT and TUM tissues was calculated as the percentage of detected FFPE probes also detected in the corresponding FF sample. (C) The fold-change correlation between FF and FFPE samples was calculated as the Log_2_ of the fold-change between the NAT and TUM tissues intensities. All detected probes (Detection p-value <0.01) are plotted. (D) Lists of differentially expressed probes were generated by comparing replicates of the NAT and TUM tissues with a false discovery rate (FDR) cutoff of <5%. The overlap of differentially expressed probes between the FF and FFPE matched samples for both the NAT and TUM tissues was calculated as the percentage of differentially expressed FFPE probes also detected in the corresponding FF sample.

We next assessed the performance of the WG-DASL assay in matched pairs of fresh frozen (FF) and FFPE samples, using RNAs derived from normal adjacent tissue (NAT) and tumor (TUM) lung tissue. We obtained a high degree of sensitivity, with the number of detected transcripts in both the NAT and TUM FFPE samples approximately 90% of that detected in the corresponding FF samples (Fig. 2A). Moreover, the overlap of the detected transcripts was also high, with 94.2% (14188/15064; NAT) and 96.3% (14066/14610; TUM) of the FFPE transcripts also detected in the corresponding FF sample (Fig. 2B). Direct intensity comparisons between matched FF and FFPE samples yielded average correlations of R^2^∼0.62; fold-change comparisons of several thousand detected transcripts between paired FF and FFPE samples were higher (average R^2^∼0.70, Fig. 2C), with a slope approximating 45 degrees and no significant compression or expansion observed for the log fold-change ratios. We also generated lists of differentially expressed transcripts between the TUM and NAT samples for both FF and FFPE tissues and then determined the extent of the overlap between the two differentially expressed transcript lists. With a false discovery rate (FDR) of <5%, approximately 74% (4793/6473) of the transcripts identified in the FFPE comparison also overlapped with those identified in the FF comparison (Fig. 2D). Similar overlapping results were obtained at a more stringent FDR (<1%) (Fig. S1).

### Fold-Change Detection and Dynamic Range

**Figure 3:**
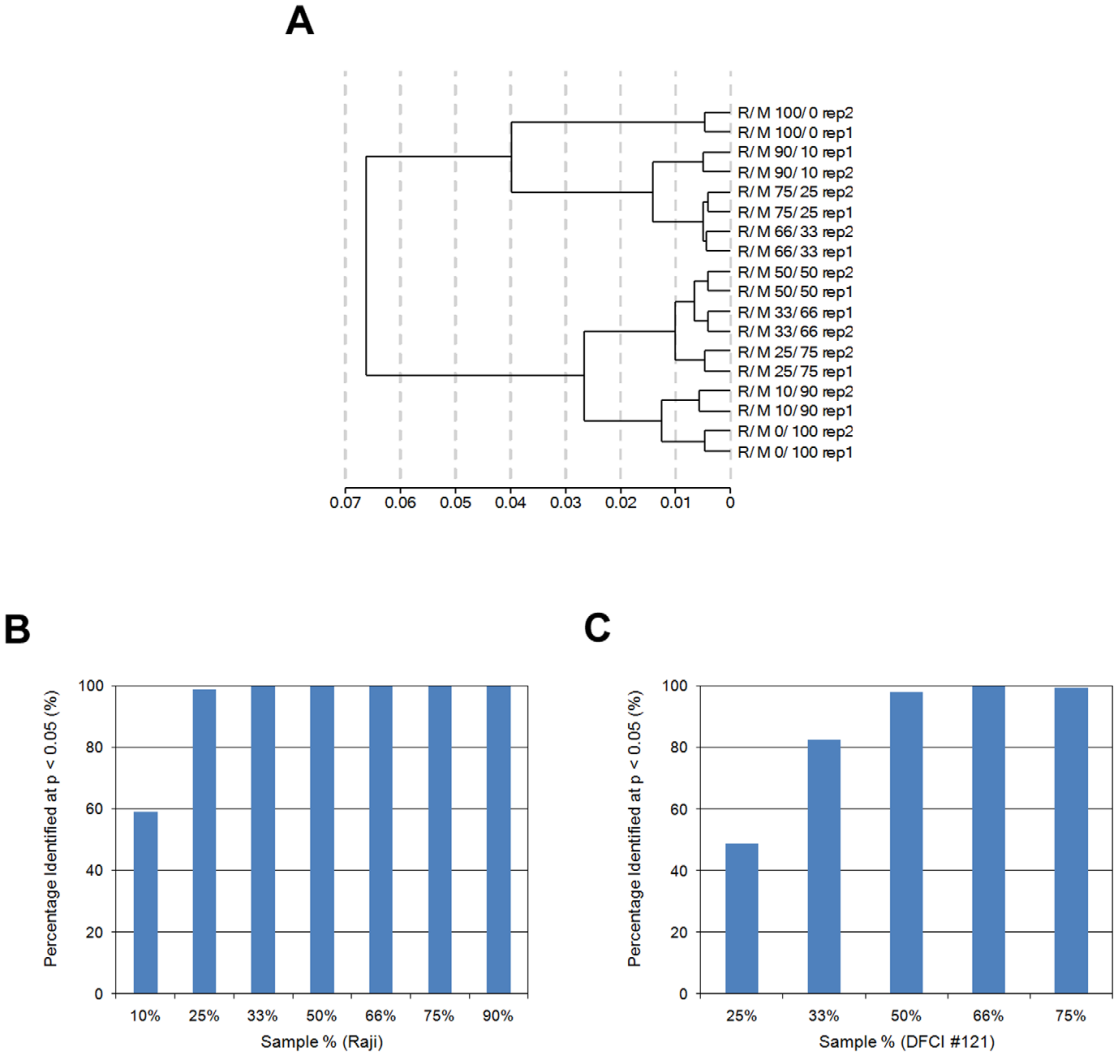
Assay fold-change detection. (A) Total RNAs from Raji (R) and MCF-7 (M) cells were mixed at various ratios, with each mixture having a combined input of 100 ng. After labeling and hybridizing, the raw data underwent unsupervised hierarchical clustering using all 24526 probes shown in the cluster dendrogram. Units on the x-axis are Pearson's correlation coefficient (R). (B) Using a set of differentially expressed probes, derived from comparisons between the two intact RNA samples (Raji and MCF-7), the number of differentially expressed probes identified in different Raji/MCF-7 mixtures was calculated and plotted as a percentage of that identified in the 100% Raji sample. Values on the x-axis correspond to the percentage of Raji RNA in each mixture, while the values on the y-axis represent the percentage of Raji-specific probes detected in each mixture at p <0.05. For example, more than 98% of the Raji-specific probes were detected in the 25% Raji mixture (1.3-fold change) and higher Raji mixtures. (C) Using a set of differentially expressed probes, obtained from comparisons between two FFPE samples (DFCI #121 and DFCI #123), the number of differentially expressed probes identified in the different DFCI #121/#123 mixtures was calculated and plotted as a percentage of that identified in the 100% DFCI #121 sample. Values on the x-axis correspond to the percentage of DFCI #121 RNA in each mixture, while the values on the y-axis represent the percentage of DFCI #121-specific probes detected in each mixture at p<0.05. For example, more than 80% of the DFCI #121-specific probes were detected in the 33% DFCI #121 mixture (1.5-fold change) and higher DFCI #121 mixtures.

To further characterize the sensitivity of the WG-DASL assay, we designed an experiment to evaluate the ability of the assay to discriminate among different RNA mixtures. Total RNAs from Raji and MCF-7 cells were mixed in the following ratios: 100/0, 90/10, 75/25, 67/33, 50/50, 33/67, 25/75, 10/90 and 0/100% (corresponding to 1.0, 1.1, 1.3, 1.5, 2.0, 3.0, 4.0 and 10.0 fold-change respectively), with six replicates performed for each mixture and having a combined input of 100 ng in each case. Unsupervised hierarchical clustering revealed that the assay was able to accurately recapitulate the expected relationships among the different mixed samples (Fig. 3A).

For a more rigorous analysis, we next selected a set of probes that were specific for the Raji cell line such that the detection p-value <0.01 for Raji, but >0.1 for MCF-7. Using these thresholds a set of 433 Raji-specific probes were obtained. The average signal intensities for this probe set in each mixture were calculated and then compared to that obtained for the 100% Raji RNA sample. With 98% of the Raji-specific probes detected in the 25–33% Raji/MCF-7 mixtures, the WG-DASL assay therefore is capable of detecting 1.3–1.5-fold changes in intact samples (Fig. 3B).

We next performed a similar experiment to assess the fold-change detection capabilities of the WG-DASL assay using FFPE samples. For this experiment RNAs from two different FFPE samples (DFCI #121 and DFCI #123) were mixed in the following ratios: 100/0, 75/25, 67/33, 50/50, 33/67, 25/75 and 0/100% (corresponding to 1.0, 1.3, 1.5, 2.0, 3.0 and 4.0 fold-change respectively), with six replicates performed for each mixture and having a combined input of 200 ng in each case. We then selected a set of probes that were specific for the DFCI #121 sample such that the detection p-value <0.01 for DFCI #121, but >0.01 for DFCI #123. Using these thresholds a set of 154 DFCI #121-specific probes were obtained. The average signal intensities for this probe set in each mixture were calculated and then compared to that obtained for the 100% DFCI #121 sample. With 82–98% of the DFCI #121-specific probes detected in the 33–50% DFCI #121/123 mixtures, the WG-DASL assay therefore is capable of detecting 1.5–2-fold changes in FFPE samples (Fig. 3C).

The WG-DASL assay also yielded a dynamic range of ∼3 logs as determined by serial dilutions of artificially synthesized RNAs spiked into a background of 100 ng total Raji RNA (Fig. S2).

### Performance with Low Inputs

**Figure 4:**
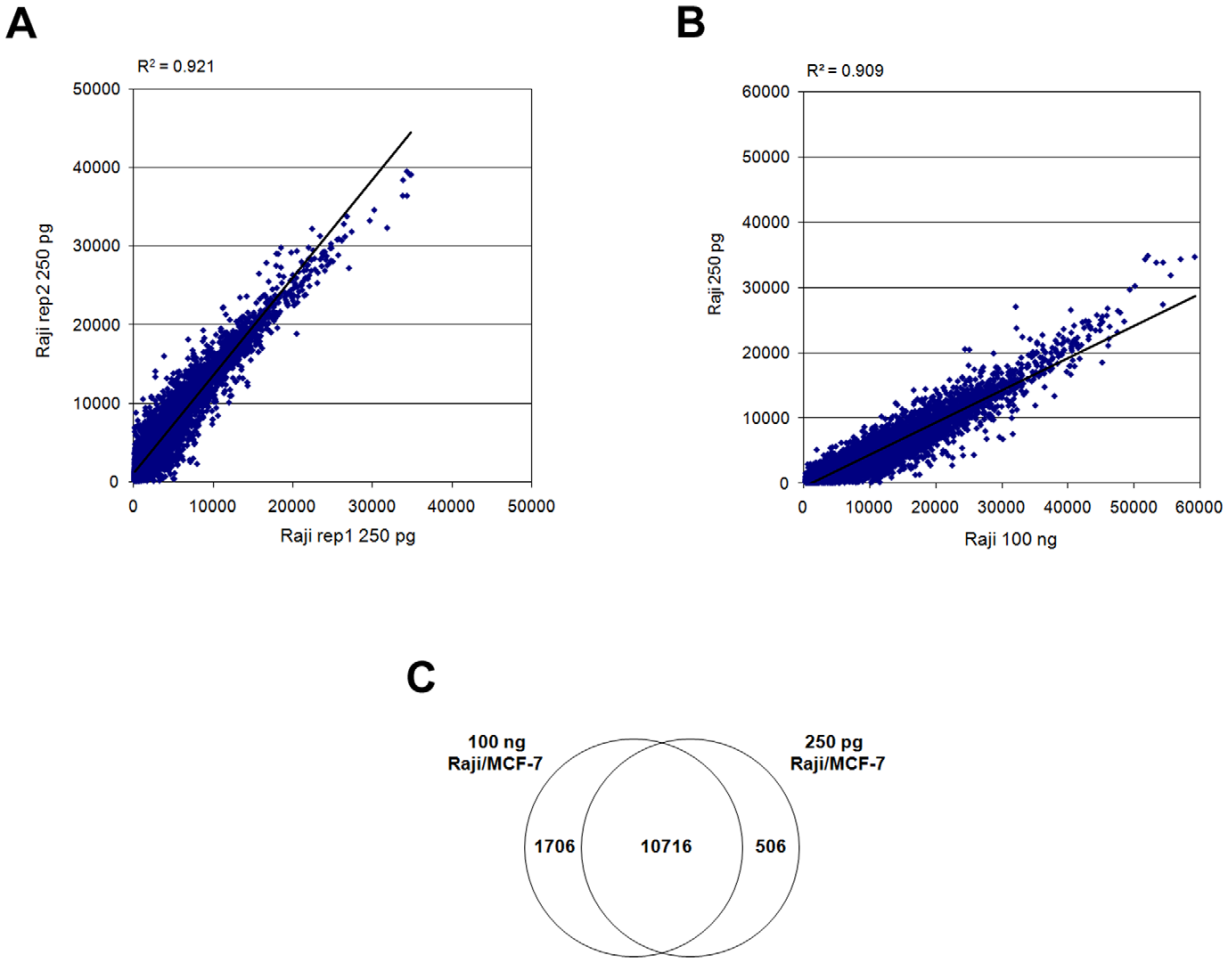
WG-DASL assay performance with low RNA inputs. (A) Raw intensity scatterplots are shown for assay replicates for 250 pg Raji RNA and (B) also for a correlation between 250 pg and 100 ng Raji RNA. (C) Lists of differentially expressed probes were generated by comparing replicate data for Raji and MCF-7 cells using a cutoff of p = 0.001. The overlap of differentially expressed probes (Raji vs. MCF-7) between the 250 pg and 100 ng RNA inputs was calculated as the percentage of differentially expressed probes identified in the 250 pg sample which was also identified in the 100 ng sample.

Having obtained robust expression profiling data with 100 ng intact total RNA, we next sought to determine the performance of the WG-DASL assay at lower RNA input levels. For this purpose various amounts (100 ng, 50 ng, 10 ng, 5 ng, 1 ng, 250 pg, 100 pg and 10 pg) of Raji and MCF-7 total RNAs were assayed. For both cell lines, data generated with as little as 250 pg still yielded reproducible expression profiles with an average correlation of R^2^∼0.92 (Fig. 4A, Table 3), while the 50 pg and 10 pg RNA inputs yielded average intensity correlations of R^2^∼0.82 and 0.78, respectively (Table 3). On average, the probe overlap as compared to 100 ng was 71.7%, 42.6% and 22.9% for the 250, 50 and 10 pg inputs respectively (Table 3). Comparison of expression profiles generated with the lower inputs to that obtained with the standard 100 ng input revealed average intensity correlations of R^2^∼0.91 for the 250 pg (Fig. 4B), and R^2^∼0.80 and 0.72 for the 50 pg and 10 pg inputs, respectively (Table 3). To further assess the ability of the WG-DASL assay to detect differentially expressed genes with lower inputs we compared lists of differentially expressed transcripts detected at the 250 pg level to that obtained with the standard 100 ng input. Using a p-value  = 0.001 cutoff, approximately 95.5% (10716/11222 probes) of the differentially expressed transcripts in the 250 pg input was also identified in the standard 100 ng input (Fig. 4C).

**Table 3:** WG-DASL Assay Performance as a Function of Intact RNA Input.

Metric	100 ng	50 ng	10 ng	5 ng	1 ng	250 pg	50 pg	10 pg
Average number of detected probes (MCF-7)	15247	15150	15060	14966	14279	10382	6671	3258
Average number of detected probes (Raji)	13291	13141	13032	13001	12197	10001	5499	3254
Average probe overlap with 100 ng (MCF-7; %)	100.0	99.4	98.8	98.2	93.7	68.1	43.8	21.4
Average probe overlap with 100 ng (Raji; %)	100.0	98.9	98.1	97.8	91.8	75.2	41.4	24.5
Average correlation with 100 ng (MCF-7; R^2^)	1.000	0.986	0.976	0.966	0.932	0.906	0.795	0.717
Average correlation with 100 ng (Raji; R^2^)	1.000	0.988	0.976	0.971	0.920	0.917	0.807	0.729
Self-Reproducibility (MCF-7; R^2^)	0.988	0.987	0.986	0.982	0.969	0.909	0.826	0.791
Self-Reproducibility (Raji; R^2^)	0.990	0.989	0.987	0.986	0.941	0.921	0.821	0.775

Values for the number of detected probes, probe overlap and correlation are derived from the average of two technical replicates. Probe overlap is calculated as a percentage of the number of probes with matching detected calls at p-value <0.01 between the low and standard inputs divided by the total number of probes detected in the standard input (100 ng). Values shown for the correlation and self-reproducibility are calculated for probes detected (p <0.01) in both inputs and replicates, respectively.

To assess the performance of the WG-DASL assay with limiting quantities of FFPE samples, we performed a similar experiment as described for the intact samples, using two FFPE samples (DFCI #120 and DFCI #129) with total RNA inputs as follows: 200 ng, 50 ng, 10 ng, 5 ng, 1 ng and 250 pg. Each sample was assayed twice. For both FFPE samples, inputs as low as 5 ng yielded detected probe concordance (overlap) rates of >85% and raw intensity correlations of R^2^>0.85 when compared with standard inputs of 200 ng (Table 1). Moreover, these results were reproducible with self-correlations of R^2^>0.90. Despite the lower probe concordance rates (∼60%) between the 1 ng input and the standard 200 ng input, the raw expression profiles at the 1 ng input level remained reproducible, with R^2^∼0.80 and had correlations of R^2^>0.75 when compared with the standard 200 ng input.

### Comparison and Validation of the WG-DASL Assay with IVT-Based Microarray Profiling and Real-Time Quantitative PCR Methods

**Figure 5:**
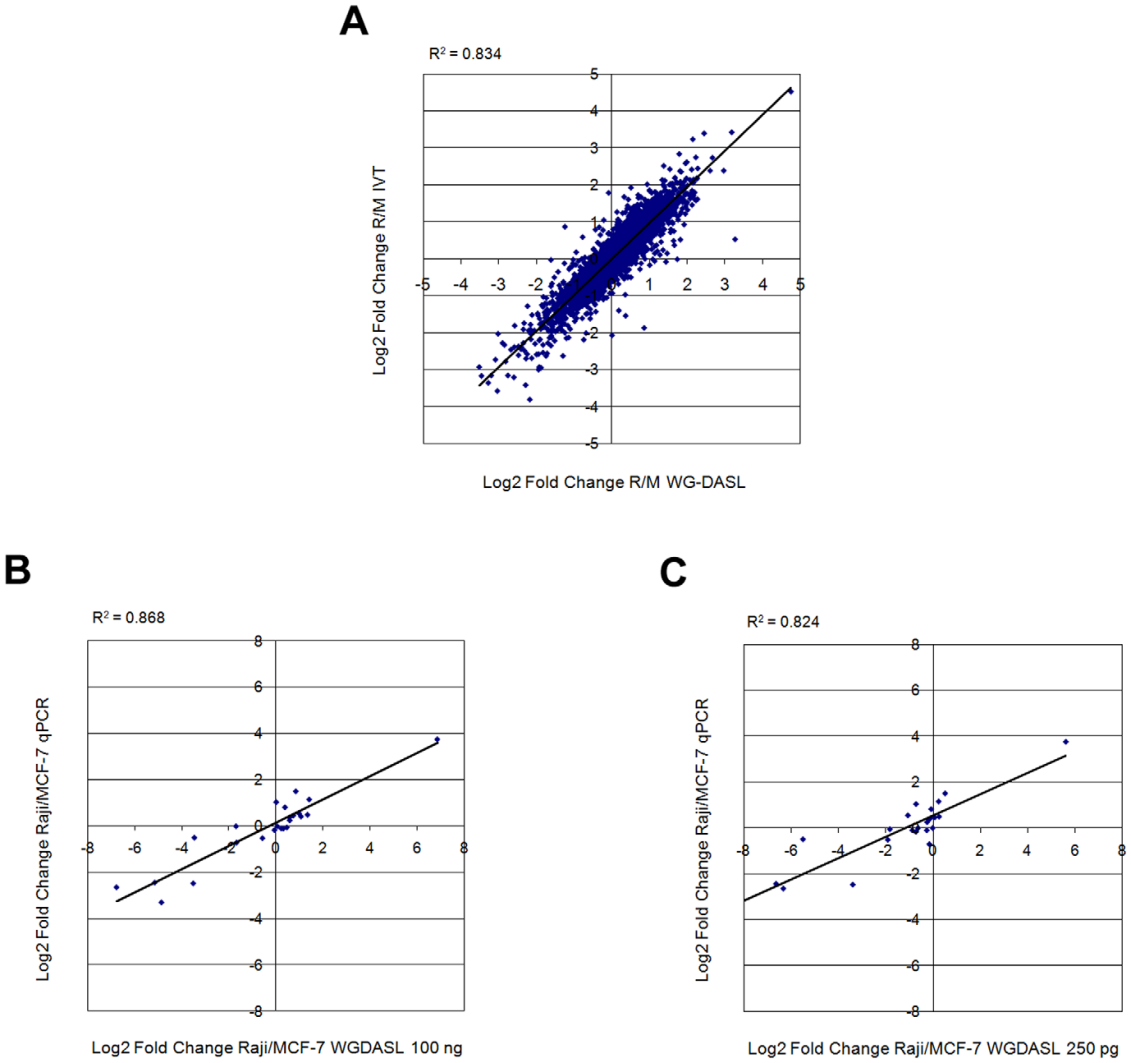
Validation with *in vitro* transcription-based and qRT-PCR assays. (A) Logarithmic fold-changes in transcript abundance between two cancer cell lines (Raji and MCF-7) were compared between the WG-DASL assay (100 ng total RNA input; x-axis) and an *in vitro* transcription (IVT)-based array platform (100 ng total RNA input; y-axis); data were quantile normalized and all common detected probes (Detection p-value <0.01) were plotted. (B) Comparison of the WG-DASL assay (100 ng total RNA input; x-axis) and qRT-PCR (y-axis) with intact RNA and (C) the WG-DASL assay with lower intact input (250 pg total RNA; x-axis) and qRT-PCR (y-axis). The qRT-PCR fold-change data are derived from Ct values for 24 common transcripts.

To validate the global expression profiles obtained with the WG-DASL assay, 100 ng total RNA derived from MCF-7 and Raji cells were labeled using an *in vitro* transcription (IVT)-based method [22] and hybridized to the same microarray. The IVT-method employed here allows linearly amplified cRNA to be directly hybridized to the same type of whole genome expression BeadChip as that used in the WG-DASL assay and provides an independent means by which different labeling methods may be compared on a global scale. Fold-change (i.e. Raji/MCF-7) data derived from both the WG-DASL assay and the IVT-method were then extracted and compared. Our analysis yielded reproducible and highly similar results, with an average correlation of R^2^∼0.80 (Fig. 5A), indicative of a high degree of measurement concordance between the two different gene expression platforms.

We also compared the WG-DASL assay results to that obtained with real-time quantitative PCR (qRT-PCR). For comparisons in intact RNAs we designed a set of primers corresponding to a panel of 24 genes, whose expression levels differed between the assayed cell lines (Raji and MCF-7). Using the “fold-change” method described earlier, our comparisons across these 24 common genes between the WG-DASL (100 ng input RNA) and the qRT-PCR assays consistently demonstrated, on average, a strong correlation (R^2^∼0.87) across replicate experiments (Fig. 5B). Furthermore, a similar degree of concordance (R^2^∼0.85) was maintained even when the WG-DASL assay was performed with 250 pg RNA input (Fig. 5C). We noted a compression of the WG-DASL fold-change data as compared to that obtained with the qRT-PCR assay – a phenomenon that has been previously reported for microarray-based expression analysis [9], [23]. For comparisons in FFPE-derived RNAs we designed a set of primers corresponding to a panel of 12 genes, with amplicon sizes ranging from 70–85 bp. We then compared WG-DASL and qRT-PCR data (Table S1) obtained for three ovarian cancer FFPE samples (DFCI #79, 98 and 118). Across the 12 assayed genes we obtained fold-change correlations of R^2^ = 0.63 for DFCI #79/98, R^2^ = 0.61 for DFCI #98/118 and R^2^ = 0.42 for DFCI #79/118, yielding an average of R^2^ = 0.55 for all three paired combinations.

## Discussion

Over the last two decades, significant strides have been made in RNA profiling in FFPE tissues, including efforts to standardize tissue handling and fixation procedures [24], [25] and improving RNA extraction methodologies for FFPE tissues [13], [26], [27]. Despite these advancements, very few technologies have emerged that are capable of robust whole transcriptome profiling in archived FFPE materials. Initial attempts at large-scale expression profiling in FFPE tissues yielded either poor reproducibility and sensitivity [28] or loss of gene signature information when compared with matched FF samples [29]. Most recently, several commercial and academic endeavors have focused on improving the efficiency of amplification and labeling of FFPE-derived RNAs and have met with varying degrees of success [5]–[8], [30]–[34]. While in some instances these technologies have demonstrated their ability to retrieve meaningful biological information from degraded samples [27], [35], concerns remain centered largely around the capability with which older archived tissues (>10 years) can be routinely profiled [29], [31] as well as reports of low signal intensities and poor (transcript) detection sensitivity in FFPE tissues [5], [7], [36].

Here we describe the development of a robust and highly sensitive profiling assay capable of generating high quality expression profiles in degraded/FFPE samples at a whole-transcriptome level. The assay is highly reproducible with as little as 50 ng total RNA extracted from FFPE tissues (R^2^>0.97). It is capable of accurately detecting 1.3–1.5 and 1.5–2 fold changes in intact and FFPE samples, respectively, with a dynamic range of 3 logs. Comparisons between matched FF and FFPE tissues yielded a high degree of profile concordance with a large number of transcripts robustly detected in both FF and FFPE samples. Detailed comparisons between normal and tumor FF and FFPE sample sets yielded significant fold-change correlations of R^2^∼0.70 and >70% of the differentially expressed transcripts shared between both FF and FFPE sample sets. For qRT-PCR vs. WG-DASL comparisons we, like others [5], also noted poorer correlations for genes with low array signal intensities, which adversely impacted the overall platform concordance. Despite this effect we obtained an average fold-change correlation of R^2^ = 0.55, a result which is consistent with that reported for other studies [5], [36]. Given its overall performance at this level, the WG-DASL assay now enables studies that previously would have been difficult, for example, using archival tissue blocks for either retrospective or prospective randomized trials. Indeed, it has already been successfully applied to interrogate global gene expression patterns in stage I serous invasive versus borderline ovarian tumors [37].

In addition to enabling whole-genome RNA profiling in FFPE tissues, the WG-DASL assay also performs well with low RNA inputs. With as little as 250 pg of intact total RNA (∼25 cell equivalent), the assay still maintains good reproducibility (R^2^ = 0.92) without significantly compromising either the intensity correlation (R^2^ = 0.91), sensitivity (>70%) or differential transcript overlap (>85%) when compared with standard amounts of RNA input (100 ng). For FFPE-derived RNAs, inputs as low as 1 ng total RNA generated reproducible expression profiles with R^2^∼0.80, while still maintaining a correlation of R^2^∼0.75 when compared with standard FFPE inputs (200 ng). Therefore, this technology is particularly useful for clinical research where minimally invasive procedures often yield limiting amounts of starting material such as fine needle biopsies and circulating tumor cells.

## Materials and Methods

### RNA Samples

Cancer cell line RNAs, breast adenocarcinoma (MCF-7) and Burkitts Lymphoma (Raji), were purchased from Applied Biosystems Inc/Ambion (Austin, TX, USA). For comparisons between fresh frozen and FFPE tissues we used matched lung tissue samples from a tumor and the normal adjacent tissue that were preserved as fresh-frozen and FFPE tissue. RNA was extracted from the fresh frozen tissue using a standard RNeasy protocol (QIAGEN GmbH, Hilden, Germany), while FFPE RNAs were isolated from 10 µm sections using the FormaPure Kit (Agencourt Bioscience Corporation, Beverly, MA, USA). The archival age for the lung FFPE tissues was 3 years. RNAs from ovarian tumor FFPE samples, with archival ages ranging from 8–13 years, were extracted using the RNeasy FFPE Kit (QIAGEN GmbH, Hilden, Germany) according to the manufacturer's instructions with a few modifications described next. Punch biopsies (3–4), 0.6 mm in diameter, were obtained from each tissue block and were placed in a tube containing 1 ml xylene. Immediately after the 100% ethanol extraction step, an additional 70% ethanol extraction was performed. Proteinase K digestions were at 55°C for 35 min followed by 80°C for 25 min. Genomic DNA was removed by centrifugation in gDNA Eliminator spin columns at 8000 x g for 1 min, after which a volume of 1400 µl ethanol was added to the flow-through fraction, resulting in three passes through the RNeasy MinElute spin column. The first Buffer RPE wash was performed at 8000 x g for 1 min and prior to the final elution the dried spin column membranes were incubated in 30 µl RNase-free water for 10 min at room temperature before centrifugation.

### Whole Genome DASL Assay Workflow

The Whole-Genome DASL assay is derived from a previously described high throughput gene expression system, the cDNA-mediated annealing, selection, extension and ligation (DASL) assay [9], but differs from the original assay by having improved multiplexing capability, thereby significantly increasing the number of transcripts that may be assayed in parallel, while still retaining the ability to profile degraded samples. Briefly, total RNA was converted to cDNA using biotinylated oligo-dT_18_ and random nonamer primers. Two assay-specific oligonucleotides were designed to interrogate a single contiguous 50 nt sequence on each cDNA. Each of these oligonucleotides consisted of two parts: an upstream-specific oligonucleotide (USO) containing a 3′ gene-specific sequence and a 5′ universal PCR primer sequence (P1), while the downstream-specific oligonucleotide (DSO) contained a 5′ gene-specific sequence and a different 3′ universal PCR primer sequence (P2′). The USOs and DSOs were designed with an average T_m_ of 65°C and 58°C, respectively and an average length of 22 and 20 nt, respectively. Using this approach, a total of 24526 oligonucleotide pairs (probes) were designed and pooled, which together constituted the whole genome DASL assay pool (DAP), corresponding to 18626 unique genes, based on well-annotated content derived from the National Center for Biotechnology Information Reference Sequence Database (Build 36.2, Release 22)). The DAP was then annealed to the targeted cDNAs during a 16 h temperature gradient (70 to 30°C) incubation followed by enzymatic extension and ligation steps, as previously described [9]. Ligated products were PCR-amplified and labeled with a universal Cy3-coupled primer after which single-stranded labeled products were precipitated and then hybridized to whole genome gene expression BeadChips as previously described [22]. BeadChips were then scanned on a BeadArray™ Reader using BeadScan software (v3.2), during which fluorescence intensities were read and images extracted. Scanned data were then uploaded into GenomeStudio® software (v1.1), via the gene expression module (WG-DASL Assay) for further analysis. All of the microarray data are MIAME compliant and have been submitted to GEO (Accession Number: GSE17599).

### Microarray Data Analysis

Unless otherwise stated, all data were analyzed in an un-normalized, raw state. All individual samples were assayed a minimum of two times and in certain instances as many as six replicates were performed. Data quality was assessed using several assay controls and Detection p-values were computed using several hundred negative controls to determine gene expression detection limits. Unsupervised hierarchical clustering was performed in GenomeStudio using Pearson's correlation coefficient and average linkage clustering. Assay performance metrics are described further in the Results section.

### Real-Time Quantitative RT-PCR (qRT-PCR)

Total RNAs were converted to cDNA using the WG-DASL assay workflow described earlier. For quantifying intact RNAs, a set of 24 primers was used to amplify approximately 200 bp fragments, while for FFPE-derived RNAs a set of 12 primers was designed to generate 70–85 bp amplicons. qRT-PCR analyses were performed on the ABI Prism 7900HT sequence detection system (Applied Biosystems Inc, Foster City, CA, USA) as described previously [9]. In brief, reactions contained 1 µl cDNA template (the equivalent of 1 ng intact RNA or 2 ng FFPE-derived RNA obtained from a serial dilution of the original WG-DASL assay-derived cDNA), 0.25 µM each of forward and reverse primer (Invitrogen, Carlsbad, CA, USA) and 5 µl SYBR Green PCR Master Mix (Applied Biosystems Inc, Foster City, CA, USA) in a total volume of 10 µl. PCR amplifications consisted of an initial enzyme activation step at 95°C for 12 min followed by 40 cycles of 95°C for 20 sec, 54°C for 20 sec and 72°C for 30 sec.

## Supporting Information

Figure S1 Differential gene expression overlap between FF and FFPE samples at FDR <1%. Lists of differentially expressed probes were generated by comparing replicates of the NAT and TUM tissues with a false discovery rate (FDR) cutoff of <1%. The overlap of differentially expressed probes between the FF and FFPE matched samples for both the NAT and TUM tissues was calculated as the percentage of differentially expressed FFPE probes also detected in the corresponding FF sample.
(6.76 MB TIF)

Figure S2 WG-DASL assay dynamic range. Intensity data from eight synthetic RNAs are shown as the number of input RNA molecules (x-axis) vs. log10 intensity (y-axis). Experiments were performed in duplicate and the error bars were calculated as the standard error of the mean.
(6.76 MB TIF)

Table S1 qRT-PCR and WG-DASL Assay Data for FFPE Tissues.
(0.07 MB DOC)
