# Supplementary figures and images for: Whole-Genome Gene Expression Profiling of Formalin-Fixed, Paraffin-Embedded Tissue Samples
Source: PLoS One. 2009 Dec 3;4(12):e8162. doi: 10.1371/journal.pone.0008162 (PMC2780295; doi:10.1371/journal.pone.0008162)

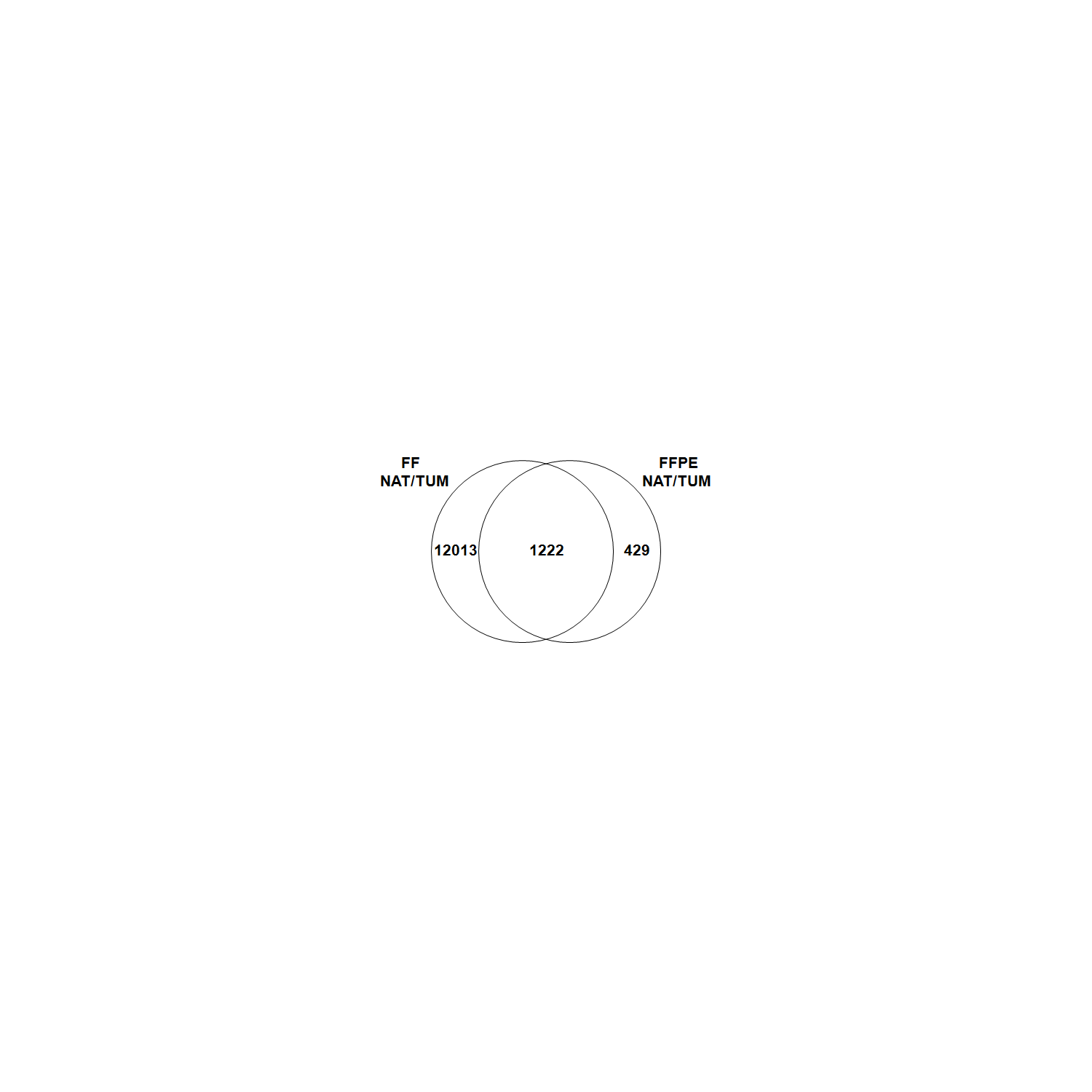

Supplement: Figure S1 — Differential gene expression overlap between FF and FFPE samples at FDR <1%. Lists of differentially expressed probes were generated by comparing replicates of the NAT and TUM tissues with a false discovery rate (FDR) cutoff of <1%. The overlap of differentially expressed probes between the FF and FFPE matched samples for both the NAT and TUM tissues was calculated as the percentage of differentially expressed FFPE probes also detected in the corresponding FF sample. (6.76 MB TIF) [file pone.0008162.s001.tif]

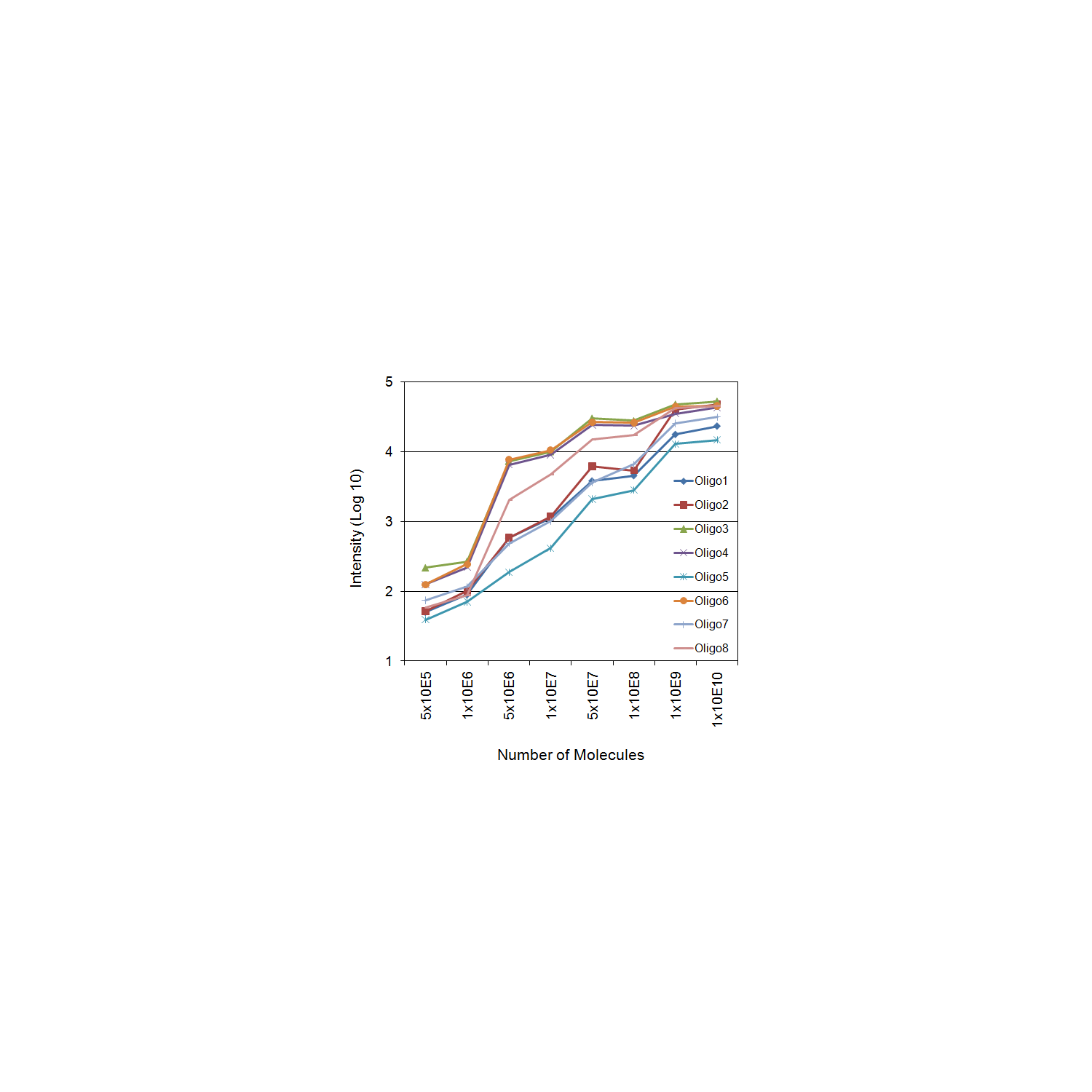

Supplement: Figure S2 — WG-DASL assay dynamic range. Intensity data from eight synthetic RNAs are shown as the number of input RNA molecules (x-axis) vs. log10 intensity (y-axis). Experiments were performed in duplicate and the error bars were calculated as the standard error of the mean. (6.76 MB TIF) [file pone.0008162.s002.tif]
