# Supplementary material for: Whole-Genome Gene Expression Profiling of Formalin-Fixed, Paraffin-Embedded Tissue Samples
Source: PLoS One. 2009 Dec 3;4(12):e8162. doi: 10.1371/journal.pone.0008162 (PMC2780295; doi:10.1371/journal.pone.0008162)
Supplement: Table S1 — qRT-PCR and WG-DASL Assay Data for FFPE Tissues. (0.07 MB DOC) [file pone.0008162.s003.doc]

| Table S1. qRT-PCR and WG-DASL Assay Data for FFPE Tissues. | | | | |  |  |  |  |
| --- | --- | --- | --- | --- | --- | --- | --- | --- |
|  |  |  | Raw qRT-PCR Ct Numbers | | | | | |
| PROBE_ID | REFSEQ_ID | GENE | DFCI #79 | | DFCI #98 | | DFCI #118 | |
|  |  |  | Replicate 1 | Replicate 2 | Replicate 1 | Replicate 2 | Replicate 1 | Replicate 2 |
| ILMN_1665004 | NM_007359.3 | CASC3 | 29.06 | 26.58 | 30.15 | 31.13 | 30.06 | 29.00 |
| ILMN_1688480 | NM_053056.2 | CCND1 | 30.17 | 30.22 | 31.27 | 32.34 | 18.83 | 30.79 |
| ILMN_1730084 | NM_007310.1 | COMT | 31.09 | 31.00 | 33.08 | 32.39 | 30.56 | 30.78 |
| ILMN_1785637 | NM_080425.2 | GNAS | 33.10 | 31.49 | 31.15 | 29.56 | 28.79 | 31.82 |
| ILMN_1807662 | NM_000876.2 | IGF2R | 29.45 | 30.68 | 31.43 | 30.49 | 31.12 | 30.28 |
| ILMN_1810214 | NM_005354.3 | JUND | 34.70 | 34.46 | 32.84 | 34.18 | 31.52 | 34.45 |
| ILMN_1652104 | NM_033360.2 | KRAS | 29.10 | 27.60 | 29.38 | 30.08 | 29.76 | 29.50 |
| ILMN_1786601 | NM_002657.2 | PLAGL2 | 28.67 | 28.69 | 30.58 | 29.62 | 29.73 | 29.15 |
| ILMN_2051519 | NM_000998.4 | RPL37A | 28.00 | 27.53 | 27.40 | 28.79 | 30.68 | 29.01 |
| ILMN_1679133 | NM_030666.2 | SERPINB1 | 29.93 | 29.53 | 32.94 | 32.04 | 32.20 | 31.44 |
| ILMN_1662438 | NM_000454.4 | SOD1 | 28.33 | 27.72 | 27.92 | 27.11 | 29.79 | 29.10 |
| ILMN_1779356 | NM_000546.3 | TP53 | 32.01 | 30.73 | 32.36 | 31.04 | 32.39 | 31.72 |
|  |  |  |  |  |  |  |  |  |
|  |  |  | WG-DASL Assay Normalized Intensities | | | | | |
| PROBE_ID | REFSEQ_ID | GENE | DFCI #79 | | DFCI #98 | | DFCI #118 | |
|  |  |  | Replicate 1 | Replicate 2 | Replicate 1 | Replicate 2 | Replicate 1 | Replicate 2 |
| ILMN_1665004 | NM_007359.3 | CASC3 | 9785 | 9397 | 5864 | 5416 | 7819 | 6777 |
| ILMN_1688480 | NM_053056.2 | CCND1 | 17367 | 16041 | 11617 | 12912 | 23204 | 22571 |
| ILMN_1730084 | NM_007310.1 | COMT | 2501 | 2979 | 2507 | 1902 | 2090 | 2753 |
| ILMN_1785637 | NM_080425.2 | GNAS | 31 | 34 | 90 | 95 | 85 | 46 |
| ILMN_1807662 | NM_000876.2 | IGF2R | 8366 | 8251 | 9148 | 8963 | 10287 | 9589 |
| ILMN_1810214 | NM_005354.3 | JUND | 20652 | 20264 | 21443 | 21536 | 22320 | 22386 |
| ILMN_1652104 | NM_033360.2 | KRAS | 49 | 44 | 46 | 43 | 47 | 56 |
| ILMN_1786601 | NM_002657.2 | PLAGL2 | 9329 | 9544 | 11625 | 12135 | 12541 | 11534 |
| ILMN_2051519 | NM_000998.4 | RPL37A | 10655 | 10337 | 7950 | 6911 | 8055 | 7682 |
| ILMN_1679133 | NM_030666.2 | SERPINB1 | 2978 | 3277 | 969 | 1215 | 1306 | 1435 |
| ILMN_1662438 | NM_000454.4 | SOD1 | 6552 | 6375 | 6838 | 6934 | 4967 | 4736 |
| ILMN_1779356 | NM_000546.3 | TP53 | 5866 | 5811 | 5567 | 5966 | 5601 | 5436 |
